# Drivers of recombinant soluble influenza A virus hemagglutinin and neuraminidase expression in mammalian cells

**DOI:** 10.1002/pro.3918

**Published:** 2020-08-14

**Authors:** Roosmarijn van der Woude, Hannah L. Turner, Ilhan Tomris, Kim M. Bouwman, Andrew B. Ward, Robert P. de Vries

**Affiliations:** ^1^ Department of Chemical Biology & Drug Discovery, Utrecht Institute for Pharmaceutical Sciences Utrecht University Utrecht Netherlands; ^2^ Department of Integrative Structural and Computational Biology The Scripps Research Institute La Jolla California USA

**Keywords:** hemagglutinin, influenza A virus, multimerization domain, neuraminidase, super folder green fluorescent protein

## Abstract

Recombinant soluble trimeric influenza A virus hemagglutinins (HA) and tetrameric neuraminidases (NAs) have proven to be excellent tools to decipher biological properties. Receptor binding and sialic acid cleavage by recombinant proteins correlate satisfactorily compared to whole viruses. Expression of HA and NA can be achieved in a plethora of different laboratory hosts. For immunological and receptor interaction studies however, insect and mammalian cell expressed proteins are preferred due to the presence of N‐linked glycosylation and disulfide bond formation. Because mammalian‐cell expression is widely applied, an increased expression yield is an important goal. Here we report that using codon‐optimized genes and sfGFP fusions, the expression yield of HA can be significantly improved. sfGFP also significantly increased expression yields when fused to the N‐terminus of NA. In this study, a suite of different hemagglutinin and neuraminidase constructs are described, which can be valuable tools to study a wide array of different HAs, NAs and their mutants.

## 1 INTRODUCTION

Influenza A virus (IAV) is a continuous burden for human and animal health, and its eradication is near impossible given the wild waterfowl reservoir. IAV contains a negative‐sense segmented RNA genome that allows for rapid nucleotide changes and exchange of whole segments both of which contribute to high variability. IAV HxNx subtypes are determined by antigenicity, however, several subtypes are under immune pressure, from which they can escape, resulting in drifted viruses. The two surface envelope proteins of IAV have opposing functions; the trimeric hemagglutinin (HA) binds to sialic acid containing glycans to enable the virus to enter cells,^1^, ^2^ the tetrameric neuraminidase (NA) cleaves sialic acids to release new viral particles from the membrane.^3^, ^4^ NA is also important for the cell entry process as it removes decoy receptors.^5^, ^6^ Both envelope proteins are therefore of great importance for the viral lifecycle and elicited antibodies impeding HA and NA biological functions and are therefore protective.^7^, ^8^, ^9^

Elucidating antigenicity, receptor specificity and other biological phenotypes of these two envelope proteins have been aided by means of recombinant soluble multimeric proteins. Also, in vaccine development and antiviral discovery, these proteins have proven to be excellent tools.^10^, ^11^, ^12^, ^13^ The use of recombinant proteins eliminates the lengthy process of virus generation either by reverse genetics or growth of wild type viruses that in turn are prone to adaptation in eggs and/or cell culture.^14^ Lab adaptation is especially problematic for older strains of influenza due to multiple rounds of infection in eggs, VERO and MDCK cells.^15^ In addition, contemporary H3N2 viruses adapt quickly to laboratory hosts.^16^, ^17^ In addition, with recombinant proteins there is no need to work in BSL‐II or ‐III environments. Individually expressed HA and NA proteins enable their functions, such as receptor specificity for HA or sialidase activity for NA, to be analyzed in great detail.

Here we report our observations gleaned over a decade of recombinant HA and NA protein expression in mammalian cells.^16^ We demonstrate increased expression yields using codon‐optimized sequences and genetic fusions of super folder GFP (sfGFP).^17^, ^18^, ^19^ Although codon‐optimization might not sound surprising, sfGFP fusions are generally utillized to facilitate routine expression and purification techniques. However, we observed a significant increase in expression yields and determined that it reduced the use of expensive antibodies and provided an excellent handle, as well as an internal read out, of a glycan binding protein. For example, we used HAs of contemporary H1 and H3 vaccine strains, the latter have been increasingly difficult to express and crystallize, most likely due to an increased number of potential N‐linked glycosylation sites that may result in an elongated retention time in the ER and Golgi.^20^ Furthermore, we applied the same principles to several NA subtypes, N1, N2, and N9. The N‐terminal sfGFP increases yields, maintains biological activity, structure and antigenicity, and aids protein quantitation during expression and purification. Our results should be valuable for other labs interested in the use recombinant HA, NA, and perhaps other viral envelope proteins.

## 2 RESULTS

### 2.1 *Codon optimization and a genetic fusion to sfGFP both increase expression yields*

**FIGURE 1:**
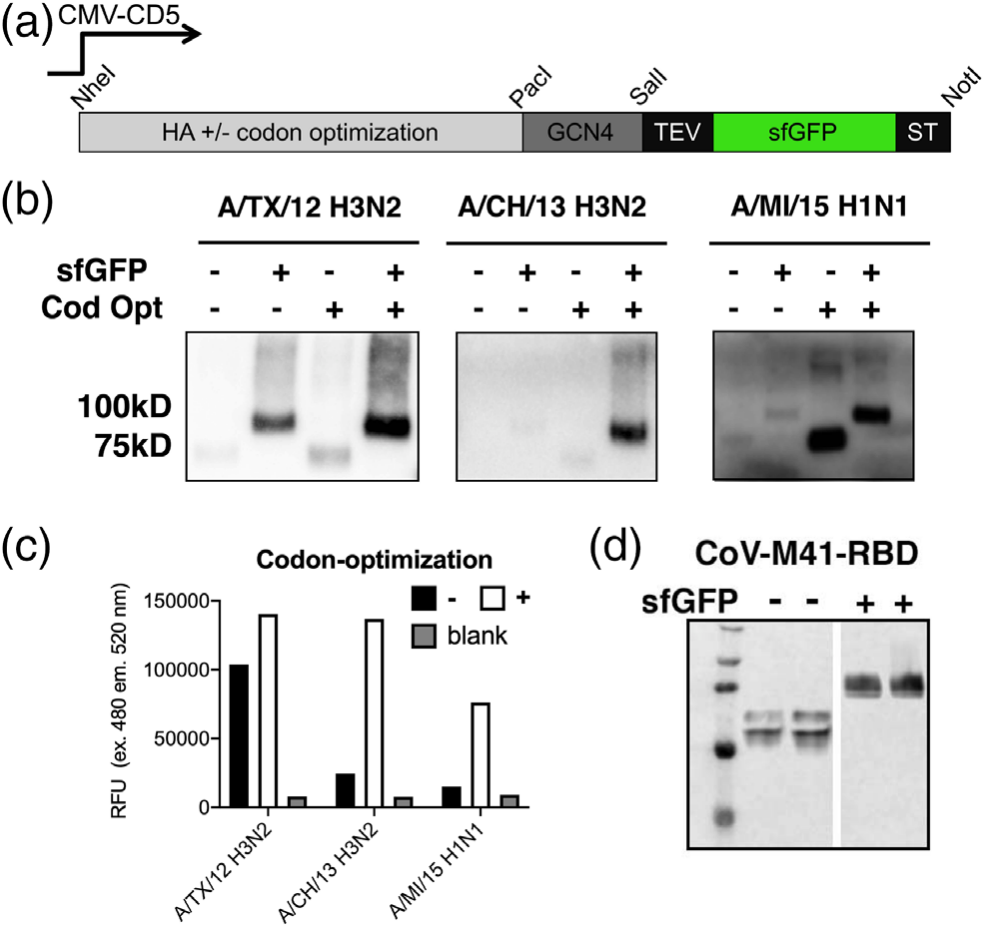
Expression yields increase by codon optimization and sfGFP fusion. (a) HA expression plasmids: Schematic representation of the HA expression cassette used. The HA ectodomain encoding sequence, under the control of CMV, was cloned in frame with DNA sequences coding for the CD5 signal peptide. C‐terminally we cloned GCN4 trimerization domain and a TEV cleavable Strep‐tag II in which we inserted a sfGFP between the TEV site and the Strep‐tag. Standard restriction sites for cloning are indicated. (b) Expression of A/TX/12, A/CH/13 H3N2, and A/MI/15 H1N1: Supernatants were analyzed by SDS–PAGE followed by western blotting, recombinant proteins were detected using a mouse HRP labelled anti‐Strep‐tag antibody. (c) Quantification: sfGFP emission was directly measured in the supernatant. Shown is a representative of four independent expression experiments over a time span of 4 weeks. (d) Avian coronavirus M41 receptor binding domain fused with sfGFP: Supernatants were analyzed by SDS–PAGE followed by western blotting, where recombinant proteins were detected using a mouse anti‐Strep‐tag antibody

Recombinant soluble HA was created with the use of an expression plasmid in which the open reading frame (orf) is preceded by a human cytomegalovirus (CMV) promoter, a CD5 derived signal peptide for efficient translation and transport to the cell culture medium (Figure 1a). The sfGFP is cleavable by a tobacco etch virus (TEV) protease recognition and cleavage site sequence. All codon optimizations, HA, NA, and sfGFP where performed by Genscripts propieratary software, standard cloning sites in the open reading frame are removed.

To demonstrate the utility of codon‐optimization for protein expression we created plasmids with and without codon‐optimized genes of contemporary H3N2 and H1N1 influenza A virus vaccine strains. We choose two H3N2 vaccine antigens, A/Texas/2012 [A/TX/12], A/Switzerland/2013 [A/CH/13], and the corresponding H1N1 A/Michigan/15 [A/MI/15]. Codon‐optimization is known to increase expression yields, whereas sfGFP fusions are not, so both sequence versions were cloned into plasmids with and without sfGFP fusions. We determined the yields by western blot and measurement of fluorescence (Figure 1b,c). All orfs were efficiently expressed when fused to a C‐terminal sfGFP and with codon optimization. While non‐codon optimized and non‐sfGFP fused variants are also observed in the cell culture supernatants, yields are less than 1 μg/ml. Using three examples, it is clear that in some cases only the addition of sfGFP or codon optimization can be sufficient to increase yields. For the A/TX/13 H3 the sfGFP increases the band intensity and incorporating a codon optimized gene further improved expression efficiency. A/CH/13 H3 HA requires both a sfGFP and codon optimization to express at high yields, whereas for the 2015 H1 vaccine component, A/MI/15 H1N1 codon optimization by itself increased expression ~10‐fold, but the addition of a sfGFP fusion did not further increase expression. We observed these expression patterns for several HA orfs and decided to show these examples, as it is increasingly difficult to express heavily glycosylated H3 HAs. As another example we cloned the receptor binding domain of an avian coronavirus into plasmids with and without a C‐terminal sfGFP. We again observed an increase of expression yield by two‐ to four‐fold as determined by band intensity (Figure 1d).

### 2.2 *N‐terminal fusions of NA*

**FIGURE 2:**
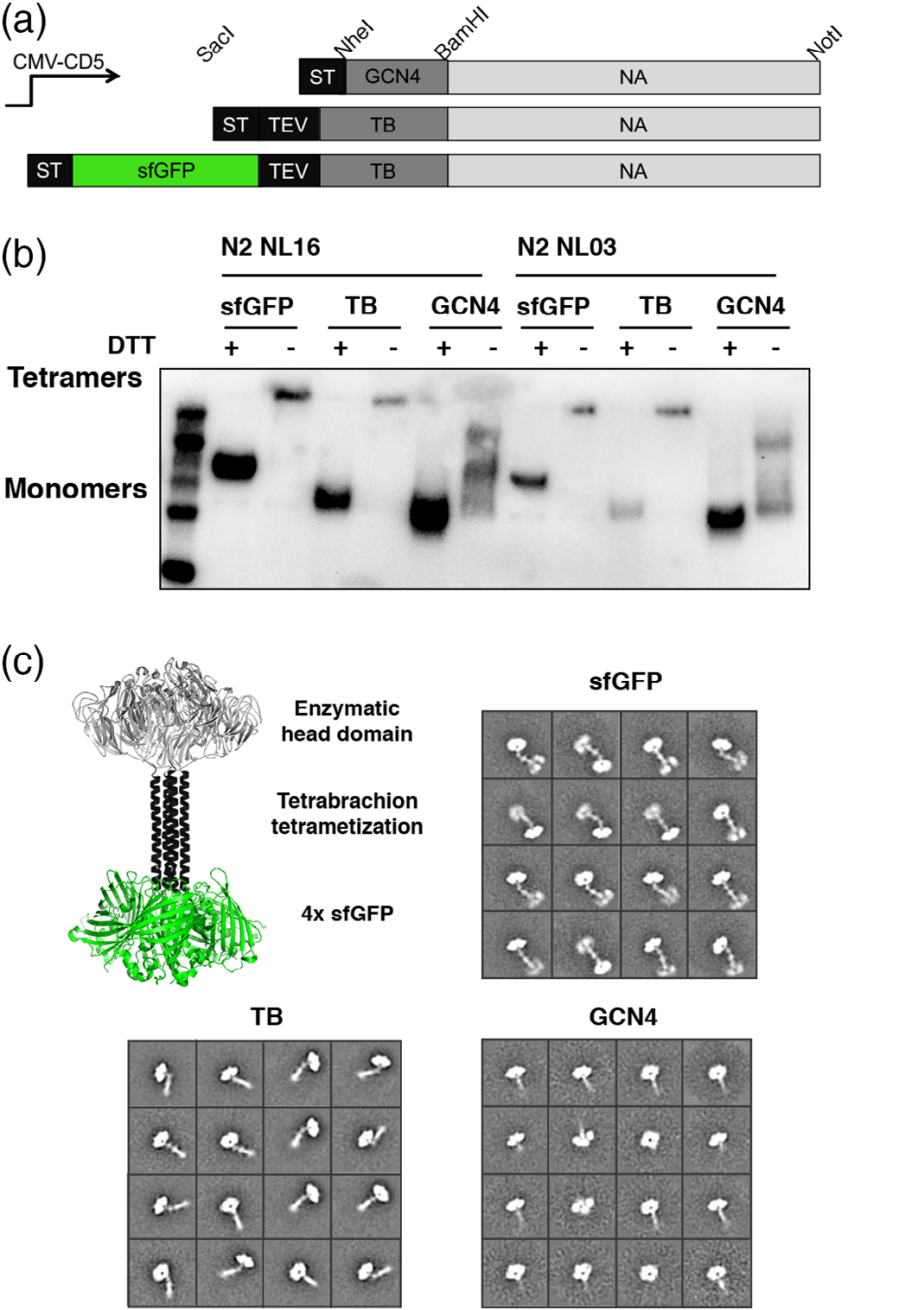
Applying N‐terminal sfGFP fusion to recombinant neuraminidase proteins. (a) Overview of created plasmids: Schematic representation of the NA expression cassettes used. The NA ectodomain encoding sequence, under the control of CMV, was cloned in frame with DNA sequences coding for the CD5 signal peptide. N‐terminally we cloned GCN4 tetramerization domain and a Strep‐tag II, a TEV cleavable Strep‐tag and a TB tetramerization domain in which we inserted a sfGFP open reading frame. Standard restriction sites for cloning are indicated. (b) N‐terminal sfGFP‐TB versus TB versus GCN4‐N2 expression: Two distinct N2 neuraminidases where expressed in HEK293T cells, and supernatants were analyzed by SDS–PAGE followed by western blotting. Supernatants were subjected on gel either non‐reduced or boiled for 5 minutes in the presence of DTT. The recombinant proteins were detected using a mouse anti‐Strep‐tag antibody. (c) Structural analyzes of NA: A structural model based on the crystal structure of neuraminidase with a tetrabrachion domain (6CRD) with four sfGFP domains added, indicating the globular enzymatic head, an extende tetramerization domain and the sfGFP fusion. Negative‐stain 2D class averages of soluble tetrameric NA proteins demonstrate that they are well folded tetramers. The N‐terminal helices and fusion proteins are visible in some class averages

Whereas HA is a Type I membrane protein, NA is Type II with its N‐terminus proximal to the membrane. Thus, we introduced the sfGFP fusion at the N‐terminus. In many expression platforms the original stalk domain is omitted^21^ as it appears to be unstable, therefore several different tetramerization domains are routinely included.^4^ To properly compare the effect of different multimerization motifs, GCN4 versus tetrabrachion (TB), and sfGFP fusion on expression, folding, and enzymatic activity, we created three different constructs (Figure 2a).^4^, ^22^, ^23^ We tested the GCN4, TB, and sfGFP‐TB‐NA proteins from two H3N2 viruses, NL/16, and NL/03. The resulting N2 proteins were analyzed by western blot where it was evident that the constructs fused to a TB domain maintain oligomerization on gel (Figure 2b). To observe monomers, we reduced the samples prior to gel loading. The GCN4 construct appeared as monomers and dimers on SDS‐PAGE. The reduced monomers of sfGFP‐TB, TB, and GCN4 migrated to different positions on gel that reflect their difference in molecular weight. In contrast to HA, we did not observe a large increase in expression yield of NA using the sfGFP fusion. We analyzed the structural arrangements of our N2 proteins to ensure they were folded into the correct tetrameric native conformation using negative stain EM, similar to our previously described sfGFP‐HA proteins.^24^, ^25^ The EM data demonstrates that the N2 NA assembles into a stable tetramer that resembled known NA structures (Figure 2c). Initially, 57,505 individual particles were picked, placed into a stack, and submitted to reference free two‐dimensional (2D) classification. From the initial 2D classes, particles that did not resemble NA were removed, resulting are final particle stacks of 32,672 particles, which were then subject to Relion 2D classification. All resultant classes demonstrated evident tetramerization and distinct NA, GCN4, and TB motifs, and four sfGFP protein structures could be identified in the EM images.

### 2.3 *Biological characterization of sfGFP‐tetrabrachion NA*

**FIGURE 3:**
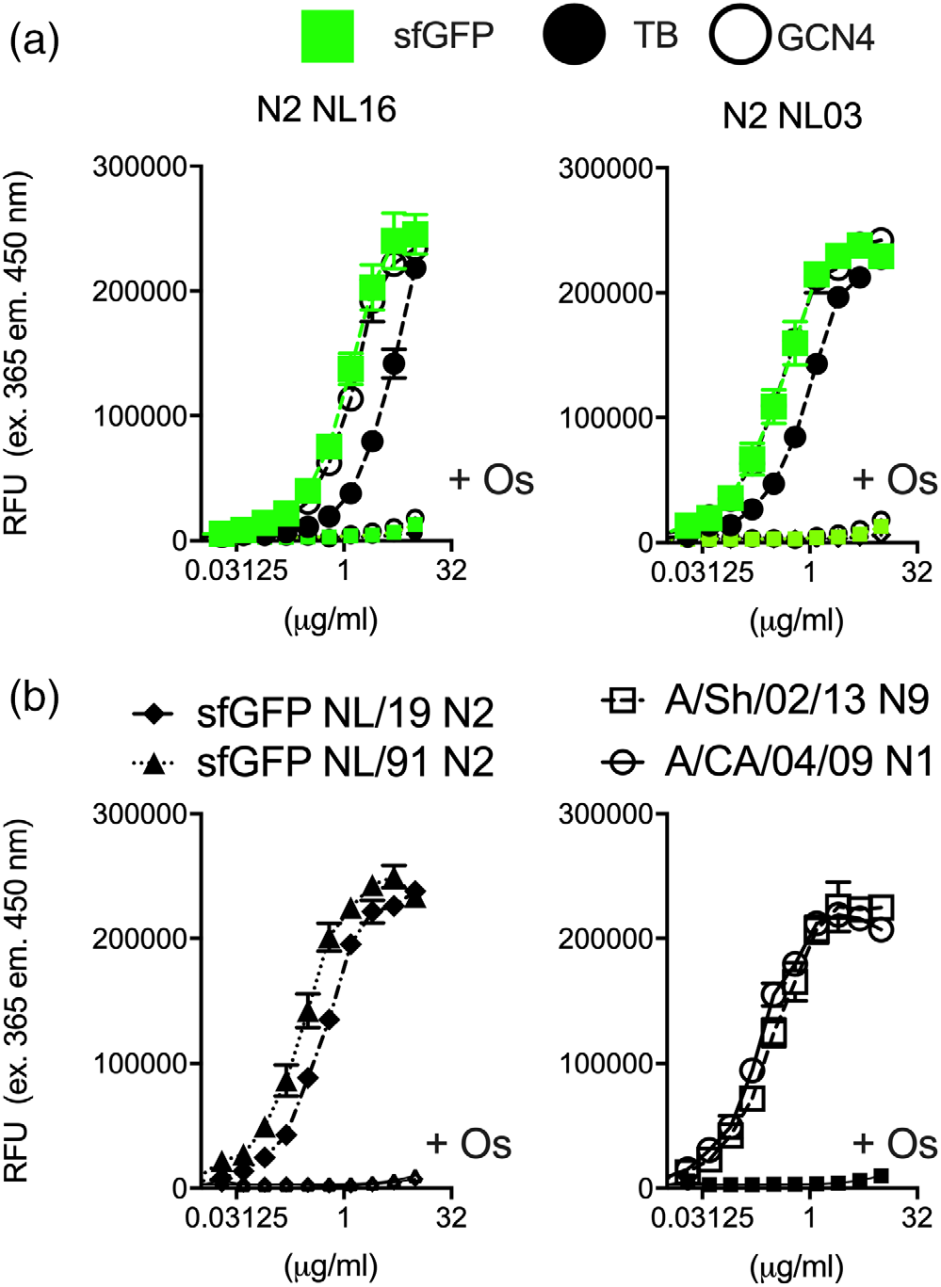
Enzymatic analyzes sfGFP fused neuraminidases. (a) Enzymatic activity of sfGFP‐TB, TB, and GCN4 Neuraminidases: NA enzymatic activity of preparations containing different amounts of the sfGFP‐TB, TB, or GCN4‐N2 was determined using the MUNANA fluorometric assay (RFU, relative fluorescent units). Oseltamivir was added to inhibit enzymatic activity. The results shown are a representative example of three independent assay performed in triplicates. (b) Enzymatic activity of sfGFP‐TB neuraminidases of different strains and subtypes: NA enzymatic activity of sfGFP‐TB N2, N9, and N1 proteins using the MUNANA fluorometric assay (RFU, relative fluorescent units). Oseltamivir was added to inhibit enzymatic activity. The results shown are a representative example of three independent assay performed in triplicates

To determine that sfGFP‐NA fusions are enzymatically, antigenically and structurally similar to their non‐fused counterparts, we analyzed the GCN4, TB, and sfGFP‐TB‐N2 proteins with MUNANA and NA specific antibodies (Figure 3). In the MUNANA assay we determined enzymatic activity by measuring methylumberriferyl, which is released from sialic acid upon digestion, as previously described.^21^, ^26^ The sfGFP‐TB‐N2 and GCN4‐N2 had the highest enzymatic activity (Figure 3a), and although TB‐N2 also displayed sialic acid cleavage, it was significantly less active. We also tested enzymatic activity of additional sfGFP‐TB NAs, two additional N2s (NL91 and NL19), an N1 from the 2009 pandemic (CA04) and the 2013 H7N9 NA (Figure 3b). All NAs tested displayed efficient sialic acid cleavage that can be inhibited by oseltamivir.

**FIGURE 4:**
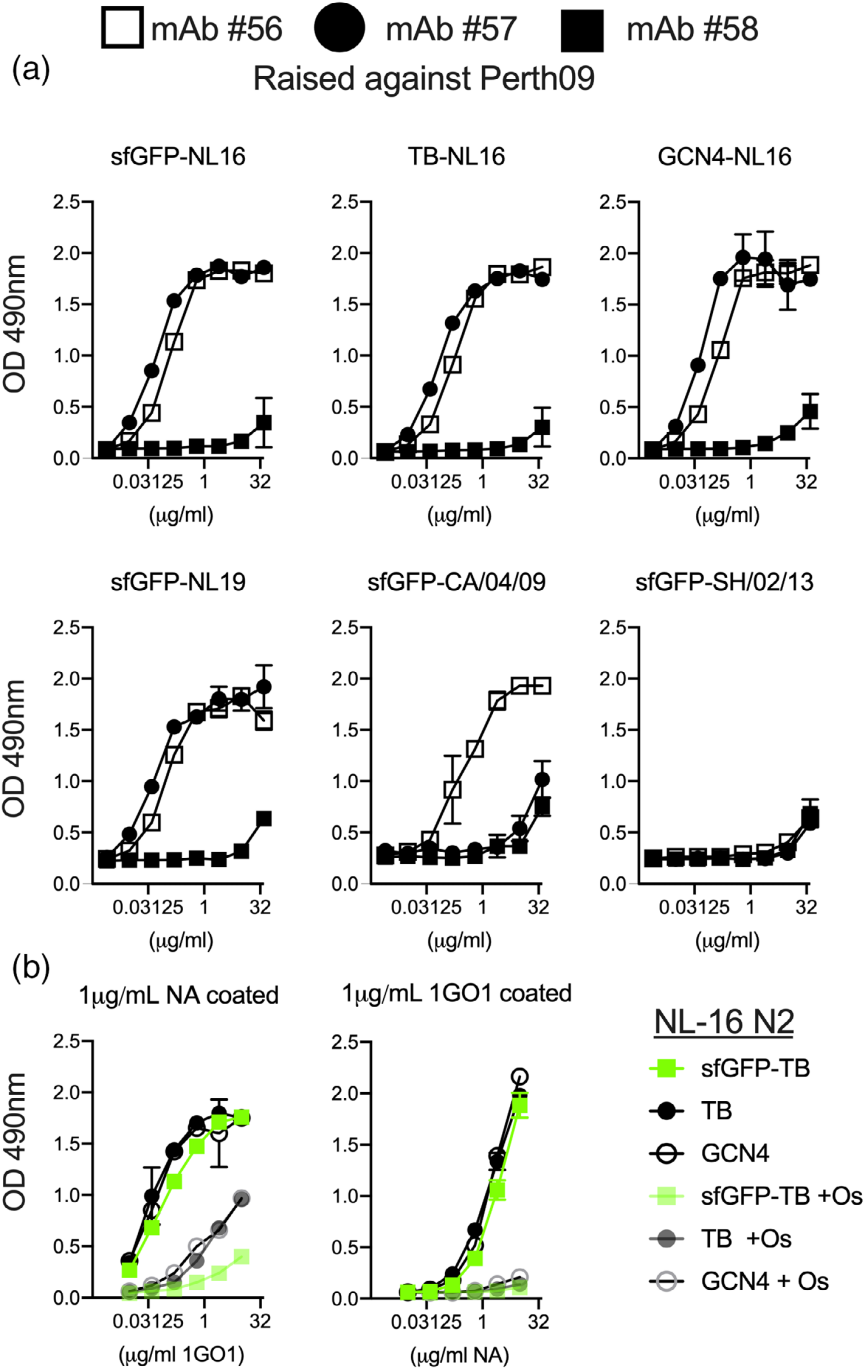
Antigenic analyzes of sfGFP fused neuraminidases. (a) Antigenicity of NA: Purified N2 proteins analyzed with three titrated mouse monoclonal antibodies. N2 proteins where coated on maxisorp 96‐wells plates and serially diluted abs were detected using a rabbit anti‐mouse. The results shown are a representative example of three independent assay performed in triplicates. (b) Pan‐NA antibody 1GO1 efficiently recognizes recombinant NAs and is inhibited by Oseltamivir: Purified NA proteins where either detected using 1GO1 (left). 1GO1 was also used capture titrated purified N2, which was detected using a mouse‐anti‐StrepTag‐HRP labelled antibody. No significant differences where observed between the differently produced NA proteins. Oseltamivir was used to inhibit the reaction 1GO1‐NA binding. The results shown are a representative example of three independent assay performed in triplicates

To analyze antigenicity, we requested three monoclonal mouse antibodies to N2 raised against Perth09 at the International Reagent Resource Program (https://www.internationalreagentresource.org/). The monoclonal antibodies were conveniently designated as #56 through #58 and binding to coated N2 proteins was analyzed using ELISA. We observed efficient binding of antibodies #56 and #57 for all sfGFP‐N2s tested, with minimal differences for N2 protein derived from viruses isolated from 1991 to 2019 (Figure 4a). Antibody #56 also bound to CA0409 N1 whereas antibody #57 did not. Ab #58 failed to bind any NA that we coated, perhaps this ab is restricted to the homologous Perth09 N2, while A/NL/16 differs by 12 amino acids. However, 2 of the 3 monoclonal antibodies had a considerable breadth across N2 strains and even N1 recognition.

Finally, we tested a recently published broadly protective NA antibody, 1G01, a kind gift of Florian Krammer,^23^, ^27^ both as a capturing as well as a detection antibody (Figure 4b). We hypothesized that direct coating of NA could potentially block the enzymatic site, which can be overcome with the bigger TB tetramerization domain in which the sfGFP is helpful to present the enzymatic sites. We therefore also coated 1G01 as a capture Ab, and applied the recombinant N2 proteins in a concentration dependent manner that where subsequently detected using the StrepTag. In both ELISA variants 1G01 efficiently recognized all N2 proteins either as detecting and capture antibody. We observed hardly any differences between the differentially expressed N2 proteins, both as a detecting as well as capture the 1G01 antibody was significantly inhibited by oseltamivir. Oseltamivir efficiently inhibited capture of N2 proteins, whereas the detection of coated N2 proteins was reduced for N2 with a sfGFP but less efficiently for GCN4 and TB only N2 proteins. Confirming our inititial hypothesis that direct coating of NA can shield the enzymatic site.

## 3 DISCUSSION

In this report we describe several optimization techniques that can substantially increase expression yields of recombinant soluble multimeric HA and NA proteins. These techniques can be extremely useful when large amounts of protein are needed, or a large number of mutants need to be expressed. We hope that our data will further enable the laboratories creating recombinant HA and NA proteins to do so in a cost‐efficient manner. We want to highlight that one of our main objectives was to minimize the expenses of expressing these complex proteins so that they can be used by labs with limited means.

Our optimization techniques ranged from a two‐ to five‐fold increases in protein expression individually, and combinations thereof could further increase yields ~10‐fold. Although our methods are generally applicable, we note that as with any approach for recombinant protein expression, results are ultimately protein dependent and can vary based on the HA or NA subtype. For example, for efficient expression of H1, the fold increases were remarkably lower compared to H3 as described in this report. Especially interesting for us was the increase in expression yield when HA or NA was fused to a sfGFP, which is in accordance with glycosyltransferases.^19^ Nevertheless, sfGFP induced increases in expression yield, is protein dependent, as for coronavirus spike receptor binding domain the increase is only two‐ to fourfold, while no increase in yields were observed for HIV env.^28^ We have not changed A206V in the sfGFP that would result in a monomeric fluorescent protein,^25^ apparently our trimerization and tetramerization domains overrule the tendency of sfGFP to dimerize. Furthermore, we have not yet used the TEV cleavage sites in these constructs, as we found that all biological properties where equal when comparing non‐ and sfGFP fused proteins.^26^ Another observation we made, is that in high salt containing buffers and elongated storage at 4°C the sfGFP dissociates, as indicated by a separate sfGFP band on gel.

Both HA and NA structures have been available for decades and now all different subtypes have been crystallized with constructs that include the TB motif, whereas multimerization motifs are normally lacking.^5^, ^27^, ^28^, ^30^ For example some labs routinely use a VASP, which perfectly amendable for crystallization after cleavage.^31^ Crystallization is however not feasible for many labs, yet structural information is in many cases vital. Low resolution structural information sufficient for mapping epitopes for antibodies can however be obtained using a relatively small amount (<10 micrograms) with the use of negative stain electron microscopy (EM). High resolution cryo‐EM structure determination requires more protein (~100 micrograms) but is sufficient to solve atomic structures such as a 3.1 Angstrom resolution structure obtained for an N9 protein fused to GCN4,^29^, ^32^ that closely resembled the negative stain image presented here.

Finally the fusion of the sfGFP facilitates several improvements in transfection efficiency, protein production determination and has been extremely useful in analyzing biological activity for HA.^24^, ^25^ We now show similar results for NA. However, the improvement of yields for NA can still necessitate expression in suspension cultures when milligram amounts are desirable. Suspension cultures, however, need expensive shaking incubators and medium. HA and NA expression in this manuscript were all done in adherent cells, with a routine milligram yield from 100 ml of supernatant for HA, making it amendable for labs with minimal means. For NA however, a 100 ml supernatant results in 250 μg. In conclusion, we demonstrate several ways to increase multimeric soluble proteins expression in mammalian cells. Which would help to increase workflow and decrease costs.

## 4 MATERIALS AND METHODS

### 4.1 *HA expression plasmid generation*

HA encoding cDNA, A/Texas/50/12 (A/TX/12 KC892248.1), A/Switzerland/9715293/13 (A/CH/13, AIU46905.1) and A/Michigan/45/15 (A/MI/15 MK622940), was synthesized by Genscript (DNA sequences available upon request), both original (a kind gift of Erhard van der Vries) and codon‐optimized sequences were cloned into the pCD5 expression as described previously.^18^ The pCD5 expression vector was adapted so that the HA‐encoding cDNAs are cloned in frame with DNA sequences coding for a signal sequence, GCN4 trimerization motif, a TEV cleavage site, a sfGFP if indicated,^25^, ^28^ and the TwinStrep, IBA, Germany). HA encoding cDNA, A/TX/50/12 (A/TX/12 genbank KC892248.1), A/CH/9715293/13 (A/CH/13) and A/MI/45/15, was synthesized by Genscript (DNA sequences available upon request), both original (a kind gift of Erhard van der Vries) and codon‐optimized sequences were cloned into the pCD5 expression as described previously.^16^ The pCD5 expression vector was adapted so that the HA‐encoding cDNAs are cloned in frame with DNA sequences coding for a signal sequence, GCN4 trimerization motif, a TEV cleavage site, a sfGFP if indicated,^24^, ^26^ and the TwinStrep, IBA, Germany).

### 4.2 *NA expression plasmid generation*

The codon optimized NA genes were synthesized at Genscript, after conventional restriction enzyme cloning,^21^ the open reading frame was preceded by sequences successively coding a Strep‐tag II and a GCN4 tetramerization domain.^33^ Additionally we cloned the NA genes in a vector adapted as such to be preceded with a sfGFP and or TB tetramerization motif.^29^ Codon optimized NA open reading frames cloned are A/NL816/91 [EPI_ISL_114608], A/NL/109/03 [EPI_ISL_113016], A/NL/354/16 [EPI_ISL_355168], A/NL/00010/19 H3N2, [EPI_ISL_336174], A/Sh/02/13 N9 [YP_009118481], and A/CA/04/09 N1 [ACP41107]. The codon optimized NA genes were synthesized at Genscript, after conventional restriction enzyme cloning,^30^ the open reading frame was preceded by sequences successively coding a Strep‐tag II and a GCN4‐pLI tetramerization domain.^34^ Additionally we cloned the NA genes in a vector adapted as such to be preceded with a sfGFP and or tetrabrachion tetramerization motif.^27^

### 4.3 *Transfecting mammalian cells*

HA and NA expression plasmids, endotoxin free, where transfected on 80% confluent HEK293S GnTI(−)cells using polyethyleneimine I (linear 25 kDa, Polysciences, Inc, Warrington, PA) (PEI) at a ratio of 1:9 g/g, before applying the DNA‐PEI mix buffered for 30 minutes in Dulbeccos Modified Eagles Medium (DMEM), 30% of the medium is removed to increase surface tension. At 6 hr post transfection, the transfection mixture was replaced with 293 SFM II expression medium (Gibco), supplemented with sodium bicarbonate (3.7 g/L), glucose (2.0 g/L), Primatone RL‐UF (3.0 g/L), glutaMAX (Gibco), valproic acid (2 mM), and DMSO (1,5%). Tissue culture supernatants were harvested 5–6 days post transfection.

### 4.4 *Determining expression yield*

HA and NA protein expression and purification was confirmed by western blotting using a StrepMAB‐HRP classic antibody. Whereas HA proteins disassociates during an SDS‐PAGE run, NA proteins need to be reduced to observe the monomeric fraction. Additionally, we measure fluorescence in the cell culture supernatant when applicable using a polarstar fluorescent reader with excitation and emission wavelengths of 480 nm and 520 nm, respectively. Proteins are purified using a single‐step with strepTactin sepharose in batch format.

### 4.5 *Negative stain EM structural analysis*

sfGFP‐TB, TB and GCN4‐PI NA proteins in 10 mM Tris, 150 mM NaCl at 4°C was deposited on 400 mesh copper negative stain grids and stained with 2% uranyl formate. The grid was imaged on a 120 KeV Tecnai Spirit electron microscope with a LaB6 filament and a 4 k × 4 k TemCam F416 camera. Micrographs were collected using Leginon^33^ and then uploaded to Appion^34^ Particles were picked using DoGPicker,^35^ stacked, and aligned using MSA/MRA.^36^ Further 2D and 3D processing was undertaken using Relion.

### 4.6 *Biological activity and antigenicity of NA proteins*

NA enzymatic activities were measured in 100 mM Tris (pH 6.15), 150 mM NaCl, 10 mM CaCl_2_ buffer, using the fluorescent substrate 2′‐(4‐methylumbelliferyl)‐α‐d‐*N*‐acetylneuraminic acid (4‐MU‐NANA) [79] with excitation and emission wavelengths of 365 and 450 nm, respectively. The reaction was conducted for 1 hr at 37°C in a total volume of 80 μl. The reactions were all performed in triplicate and were stopped by adding 80 μl of 1 M Na_2_CO_3_.

ELISAs were coated with 1 μg/ml N2 protein in PBS on maxisorp 96‐wells plates over night at 4°C. Plates were blocked for 1 hr at RT with 1% BSA in PBS supplemented with 0.1% Tween20. Mouse monoclonal antibodies were serially diluted and incubated for 1 hr at RT. Primary antibody binding was detected with a secondary rabbit anti‐mouse HRP antibody (Novus) at 1:2,000 for 1 hr at RT and as an HRP substrate, sigma fast ODP tablets were used. A similar procedure was used when 1G01 abs were coated, after blocking, the recombinant NA proteins were serially diluted and detected with a mouse anti‐Streptag HRP labelled antibody at a 1:2,000 dilution. The HRP reactions were stopped after 3 min using 2,5 M H_2_SO_4_. Optical density was measured at 490 nm, all assays were performed in triplicates and a representative result is shown from three independent biological repeats.

## AUTHOR CONTRIBUTIONS

**Roosmarijn van der Woude:** Data curation; investigation; methodology. **Hannah L. Turner:** Data curation; formal analysis; methodology; writing‐original draft. **Ilhan Tomris:** Conceptualization; investigation; methodology. **Kimberly M. Bouwman:** Data curation; supervision; writing‐original draft. **Andrew B. Ward:** Conceptualization; data curation; funding acquisition; project administration; resources; supervision; validation; visualization; writing‐original draft; writing‐review and editing. **Robert P. de Vries:** Funding acquisition; investigation; methodology; project administration; supervision; writing‐original draft; writing‐review and editing.
